# Free Radical Scavenging Activity and Characterization of Sesquiterpenoids in Four Species of *Curcuma* Using a TLC Bioautography Assay and GC-MS Analysis

**DOI:** 10.3390/molecules15117547

**Published:** 2010-10-27

**Authors:** Jing Zhao, Jiang-sheng Zhang, Bin Yang, Guang-Ping Lv, Shao-Ping Li

**Affiliations:** 1 Institute of Chinese Medical Sciences, University of Macau, Macao SAR, China; 2 Institute of Chinese Materia Medica, Chinese Academy of Chinese Medical Sciences, Beijing, China

**Keywords:** *Curcuma*, TLC, DPPH, GC-MS, Free radical scavenging activity

## Abstract

The sesquiterpenoids are one of major groups of antioxidants in *Curcuma* besides curcuminoids. However, the real substances contributing to the antioxidant activity are still unknown. In this paper, the antioxidant activity of sesquiterpenoids in four species and two essential oils from *Curcuma* genus was determined and compared based on TLC separation and DPPH bioautography assay. Their antioxidant capacities were quantitatively evaluated using densitometry with detection at 530 nm (λ_reference_ = 800 nm) using vitamin C as reference. The results showed that *Curcuma longa* rhizomes had the highest antioxidant capacity while *C. phaeocaulis* presented the lowest one among the four species of *Curcuma*. Moreover, essential oil of *C. wenyujin* showed higher antioxidant potential than that of *C. longa*. The main TLC bands with antioxidant activity of the four species of *Curcuma* were collected and characterized using GC-MS, and thus curzerene, furanodiene, α-turmerone, β-turmerone and β-sesquiphellandrene were determined as major sesquiterpenoids with antioxidant activity in *Curcuma*.

## 1. Introduction

*Curcuma* belongs to the family Zingiberaceae, some of which have been used as traditional Chinese medicines for a long time. Generally, the rhizomes of *Curcuma phaeocaulis* Val., *C. kwangsiensis* S.K.Lee & C.F.Liang, *C. wenyujin* Y.H.Chen & C.Ling and *C. longa* L. are, respectively, used as *Ezhu* or *Jianghuang* according to the Chinese Pharmacopoeia (2010 edition) [1]. The antioxidant activities of essential oils form *C. wenyujin* and *C. longa* have been reported [2,3]. However, the real substances contributing to the antioxidant property of essential oils from curcuma, which contain a large amount of sesquiterpenoids [4,5], are still unknown, and should be elucidated. 

A cardinal property of an antioxidant is the ability to scavenge free radicals. The DPPH radical is extensively used for assessing radical scavenging [6,7,8,9]. Consolidating chromatographic separation and free radical scavenging activity determination allows one to evaluate the antioxidant activity of each component in the whole extract. Though a technique based on measuring the radical scavenging activity of individual compounds using on-line HPLC method has been described [10,11], the on-line system may have slow reaction kinetics that give an inaccurate characterization of active compounds [12]. Especially, the effects of herbal extracts may contribute to the integrated activity of multiple trace components with weak biological activity, and HPLC with high resolution is not available for screening these compounds. Indeed, no obvious antioxidant was found in essential oil of *C. wenyujin* by using online HPLC coupled with an ABTS-based assay [13]. TLC combined with DPPH bioautography assay *in situ* is a method has the ability to ensure the sufficient reaction and group isolation of individual components in the mixture. The active compounds were seen as clear spots against a purple background on TLC plate [14,15,16,17]. It also allows further isolation and characterization of the compounds by using high resolution chromatographic such as HPLC analysis [18].

In this paper, sesquiterpenoids of four species of *Curcuma* were separated and their antioxidant activities were determined *in situ* based on TLC separation and a DPPH bioautography assay. The compounds in the active bands on TLC were collected and further analyzed by using GC-MS. The antioxidant capacities of the four species of *Curcuma* were also compared using vitamin C as reference.

## 2. Results and Discussion

### 2.1. Optimization of method

**Figure 1:**
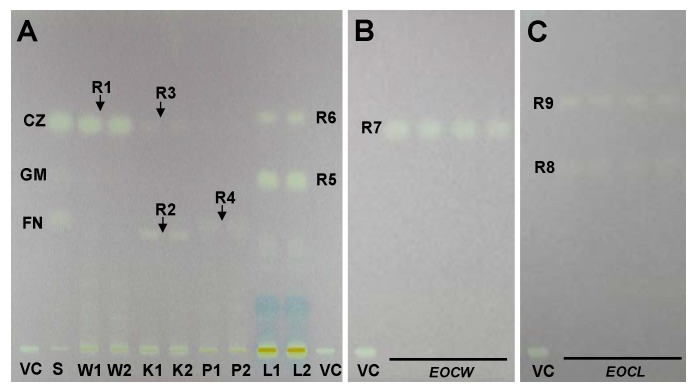
Typical TLC photography of methanol extracts of rhizomes from four species of *Curcuma* (A), essential oils of *C. wenyujin* (B, EOCW) and *C. longa* (C, EOCL) colorized with 0.04%DPPH.

Petroleum ether-ethyl acetate (90:10, v/v) provided for separation of low polarity compounds in *Curcuma* with good resolution at one run [19]. The results showed that sesquiterpenoids in four species of *Curcuma* were well separated on TLC (Figure 1). 

**Figure 2:**
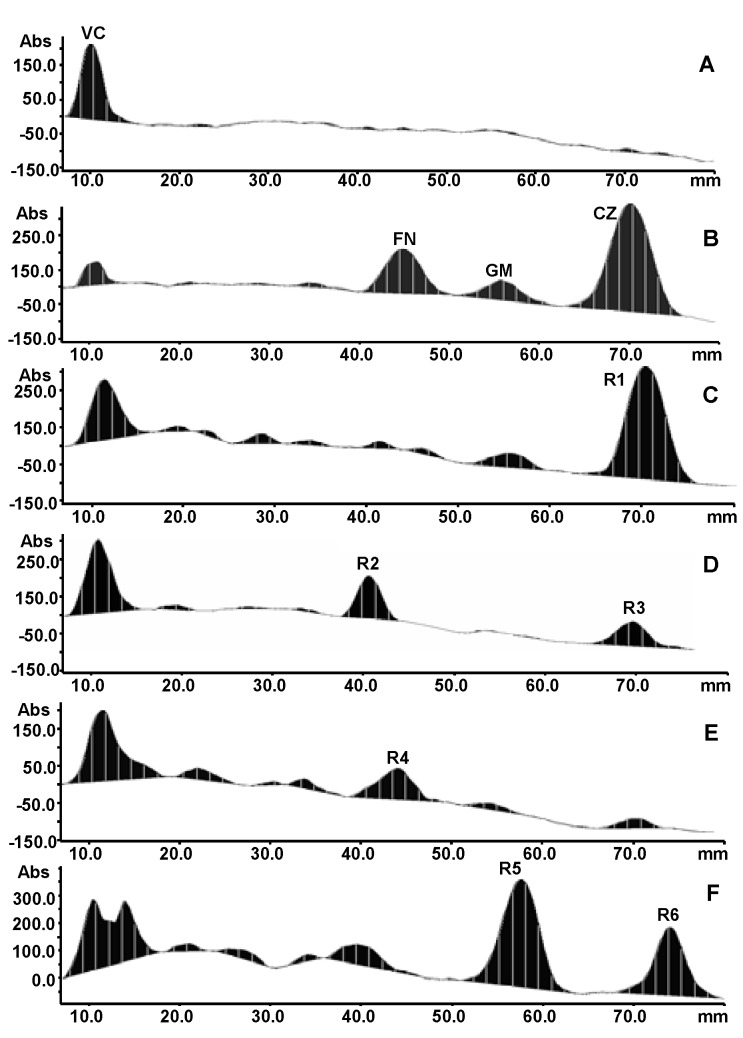
Typical TLC densitograms of vitamin C (A), mixed standards (B), methanol extracts of *C. wenyujin* (C), *C. kwangsiensis* (D), *C. phaeocaulis* (E) and *C. longa* (F) colorized with 0.04%DPPH.

The detection wavelength for quantification of VC was selected based on its absorption spectra. As a result, 530 nm was selected for evaluation of antioxidant activity of VC (Figure 2).

### 2.2. Validation of the method

#### 2.2.1. Calibration curve for antioxidant capacity of vitamin C

Standard solutions of vitamin C at a series of amount (0.5, 1.5, 2.5, 3.5, 4.5, 5.5, 6.5, 7.5, 8.5 and 9.5 μg) were used to test the linearity of its antioxidant capacity. The calibration curve was constructed by plotting the peak areas versus amount of the analyte. The linearity equation is *y* = 71.21*x* + 861.59 with R^2^ ≥ 0.9951, linear range was 1.5-7.5 μg.

#### 2.2.2. Stability

A certain amount of vitamin C standard solution was analyzed by TLC method and colorized with DPPH bioautography assay as mentioned above. The bands were scanned at 530 nm and the band peak area was determined at every 10 min in 2 hours. RSD was calculated to evaluate the stability of colorization. The results showed that the peak area decreased with time extended, but RSD was less than 4.9% in 2 hours.

#### 2.2.3. Repeatability

The repeatability was evaluated by preparing and analyzing six individually prepared solutions of the same sample (0.5 g each). The active bands of R1 (*C. wenyujin*), R2 (*C. kwangsiensis*), R4 (*C. phaeocaulis*), R5 and R6 (*C. longa*) were used as markers for evaluating the repeatability because no sample contained all antioxidant bands. One spot of each solution was analyzed on the same plate, and RSDs of the investigated bands were calculated based on the same sample solutions, which were 1.9 %, 3.5%, 3.7%, 3.8% and 4.0% for R1, R2, R4, R5 and R6, respectively. 

### 2.3. TLC bioautography assay 

After separation on TLC plates, the compounds with radical scavenging activity were determined in situ by DPPH bioautography assay. As shown in Figure 1, the yellowish white bands indicated they had antioxidant activity. The intensity showed the antioxidant capacity in the samples. 

### 2.4. Identification of compounds in active bands

Curzerene (CZ), germacrone (GM) and furanodienone (FN), which are known antioxidants, were used as reference for the primary screening of TLC analysis. The compounds in main active bands of four species of *Curcuma* were subsequently confirmed by GC-MS. All the components in investigated bands were completely separated and identified according to the mass spectra by comparing with references [20,21]. The MS data and identified compounds were shown in Table 1. The results showed that curzerene and furanodiene were the major sesquiterpenoids with antioxidant activity in *C. wenyujin*, and *α*-turmerone, *β*-turmerone and *β*-sesquiphellandrene were the major ones in *C. longa*.

### 2.5. Comparison of antioxidant capacities of four species of Curcuma

Antioxidant ability of each band was evaluated with vitamin C as reference, which gives the amount that corresponds to vitamin C. The total amount of each sample was used to evaluate their antioxidant capacities. The results were shown in Table 2. In summary, the antioxidant activities of sesquiterpenoids in rhizomes of four species of *Curcuma* were obviously different. *C. longa* gave the highest activity and *C. phaeocaulis* had the lowest one. The essential oils of *C. wenyujin* (759.3 mg/g *vs.* 7.5-15.8 mg/g) and *C. longa* (373.1 mg/g *vs.* 10.6-13.1 mg/g) showed much higher antioxidant activities than their related raw materials, which suggested that essential oil with antioxidant sesquiterpenoids were, at least, one of their major antioxidant compounds.

## 3. Experimental

### 3.1. Materials 

**Figure 3:**
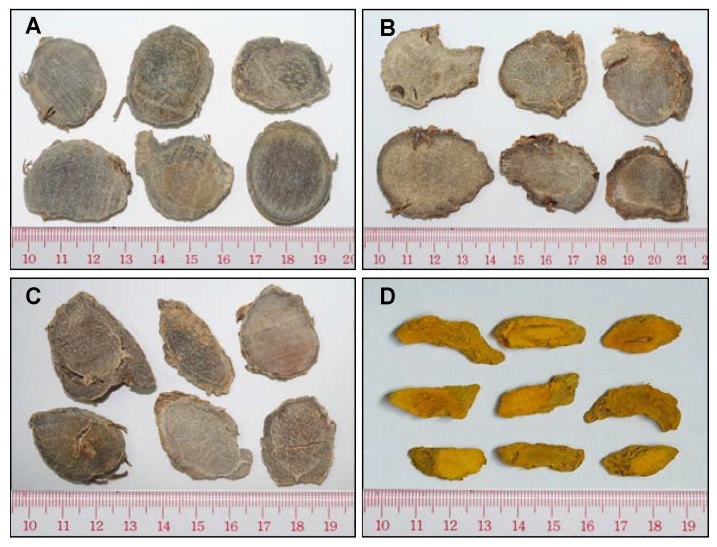
The sliced raw materials of *C. kwangsiensi* (A), *C. phaeocaulis* (B), *C. wenyujin* (C) and *C. longa* (D).

The rhizomes of *C. wenyujin*, *C. phaeocaulis*, *C. kwangsiensis* and *C. longa* were collected from different provinces of China (Table 3). The botanical origin of materials was identified by corresponding authors and the voucher specimens were deposited at the Institute of Chinese Medical Sciences, University of Macau, Macao, China. The fresh materials were carefully cleaned and cut into slices, then dried at 40 °C (Figure 3). Dried slices were ground and sieved to obtain 40-120 mesh powder. Essential oils of *C. wenyujin* (EOCW) and *C. longa* (EOCL) were purchased from Zhejiang TianRui Pharmacological Limited Company and Sichuan Xinshuchuang Development Limited Company, respectively.

### 3.2. Chemicals

All solvents and reagents (analytical grade) were obtained from UNI-CHEM d.o.o. (Belgrade, Serbia and Montenegro). Deionized water was prepared by Millipore Milli Q-Plus system (Millipore, Billerica, MA, USA). Germacrone, curcumenone, *β*-elemene, α-turmerone, *β*-turmerone, furanodienone, furanodiene and curzerene were separated from commercial oil of *C. wenyujin* and *C. longa* in our lab, and the structures were confirmed by their UV, MS and NMR data, purity of all compounds were > 99% as measured by HPLC and/or GC [20,21]. Vitamin C (VC) and 2,2′-diphenyl-1-picrylhydrazyl (DPPH) were purchased from Sigma Aldrich (St. Louis, MO, USA). 

### 3.3. Standard and sample preparation 

Positive control solution was prepared by dissolving VC (1 mg) in methanol (2 mL). Mixed standards solution was obtained by mixing 200 μL curzerene (4.4 mg/mL) solution, 300 μL germacrone (4.5 mg/mL) solution and 300 μL furanodienone (4.9 mg/mL) solution. The dried rhizome powder (approximately 0.5 g) of *Curcuma*, accurately weighed, was mixed with methanol (5 mL) in a sealed tube. The solution was treated in an ultrasonic clean bath (Bransonic, Danbury, CT, USA) for 60 min (881 w, 43 kHz) at room temperature (25 ± 2 °C). After centrifugation in an Allegra X-15R refrigerated centrifuge (Beckman Coulter, Fullerton, CA, USA) for 10 min (4500 rpm), the supernatant was colleted for TLC analysis. Solutions of essential oils of *Curcuma* were prepared by dissolving the oil (4.6 mg) in methanol (1 mL).

### 3.4. TLC analysis

TLC was performed on silica gel 60F_254_ TLC plates (Merck, Darmstadt, Germany), and a HPTLC system (Desaga GmbH, Germany) including a AS30 HPTLC applicator and CD 60 HPTLC densitometer equipped with Pro Quant Windows software. Positive control solution (5 μL) and mixed standard solution (10 μL) as well as *Curcuma* sample solution (8 μL) were spotted in duplicate on the plate as bands 9 mm wide, 7 mm apart and 10 mm from the bottom edge, respectively. The plate was developed to a distance of 75 mm with petroleum ether-ethyl acetate (90:10, v/v) in a Desaga 20 cm × 20 cm glass flat-bottom chamber after equilibration with mobile phase vapor for 30 min. The developed plate was dried in an aerator at room temperature, and then colorized by spraying with 0.04% DPPH-methanol solution (20 mg DPPH dissolved in 50 mL methanol) and heated at 40 °C on a YOKO-XR plate heater (Wuhan YOKO Technology Ltd., China) for 60 min. The treated plates were scanned at λs (scan wavelength) = 530 nm and λr (reference wavelength) = 800 nm in reflectance-extinction mode by use of the densitometer. The source of radiation was a tungsten/deuterium lamp. The slit dimensions were 6.00 nm × 0.02 nm. The negative peak was shown as signal, and the peak area was used to evaluate the antioxidant capacity of the band using VC as reference. The contribution ratio of the band to antioxidant capacity of *Curcuma* was calculated as the percentage of each peak area to the total value of area.

### 3.5. Characterization of antioxidants 

The TLC bands (located with the parallel lane colorized with DPPH solution) with antioxidant activity of four species of *Curcuma* were scraped from plates, then mixed with 1 mL ethyl acetate in a sealed tube. The mixture was treated in an ultrasonic water bath (881 w, 43 kHz) for 10 min. After centrifugation in an Eppendorf centrifuge 5415D (Eppendorf, Hamburg, Germany) for 3 min (13,000 rpm), the supernatant was collected for GC-MS analysis.

GC–MS was performed on an Agilent 6890 gas chromatography instrument coupled with an Agilent 5973 mass spectrometer and an Agilent ChemStation software (Agilent Technologies, Palo Alto, CA, USA). A HP-5MS capillary column (30 m × 0.25 mm i.d.) coated with 0.25 μm film (5% phenylmethylsiloxane) was used for separation. The mass spectrometer was operated in electron-impact (EI) mode, the scan range was 40–550 amu, the ionization energy was 70 eV and the scan rate was 0.34 s per scan. GC-MS analysis of *Ezhu* (rhizomes of investigated species of *Curcuma* except *C. longa*) samples was performed according to reported method [20] with some modification. In brief, inlet mode and temperature was pulsed splitless at 190 °C. The column temperature was set at 60 °C and held for 2 min for injection, then programmed at 5 °C/min to 120 °C and held for 13 min at temperature of 120 °C, then programmed at 25 °C/min to 145 °C and held for 25 min at temperature of 145 °C, then at 5 °C/min to 200 °C, and finally, at 20 °C/min to 280 °C, and held for 3 min at temperature of 280 °C. High purity helium was used as carrier gas of 1.0 mL/min flow-rate. The quadrupole, ionization source temperature of MS were 150 °C and 280 °C, respectively. For GC-MS analysis of *Jianghuang* (rhizome of *C. longa*) [21], the column temperature was set at 80 °C for injection, then programmed at 20 °C/min to 120 °C, then at 1 °C/min to 130 °C and held for 5 min, then at 4 °C/min to 160 °C, finally, at 20 °C/min to 280 °C. Split injection (2 μL) was conducted with a split ratio of 10:1 and high purity helium was used as carrier gas at 1.0 mL/min of flow-rate. The inlet, ionization source temperature of GC-MS were 250 °C and 280 °C, respectively. 

## 4. Conclusions 

TLC bioautography method combined with GC-MS analysis were applied for screening and identifying antioxidants in four species of *Curcuma*. Curzerene, furanodiene, *α*-turmerone, *β*-turmerone and *β*-sesquiphellandrene were found as antioxidants in *Curcuma*. Essential oils of *C. wenyujin* and *C. longa* had strong antioxidant activity.

## Figures and Tables

**VC,** Vitamin C; **S**, mixed standards; **CZ**, curzerene; **GM**, germacrone; **FN**, furanodienone; **R1-R9**, the bands with obvious antioxidant activity; **W1, W2**, two selected samples of *C. wenyujin*; **K1, K2**, two selected samples of *C. kwangsciensis*; **P1, P2**, two selected samples of *C. phaeocaulis*; **L1, L2**, two selected samples of *C. longa*.

**R1-R6**, the same as in Figure 1.

**Table 1:** The compounds in the active bands that contribute to antioxidant activity of the tested samples.

Bands ^a^	Samples	Mass Data	Compound
R1, R3, R7	*C. wenyujin EOCW*	216 (M+, 19), 201 (12), 148 (38), 108 (100), 93 (12), 91 (18), 79 (17), 77 (16)	curzerene
R1, R3, R7	*C. wenyujin EOCW*	216 (*M*+, 47), 201 (16), 159 (30), 145 (30), 108 (100), 91 (38), 77 (26), 65 (12), 53 (13), 41 (17)	furanodiene
R2	*C. kwangsciensis*	232 (M+, 100), 203 (27), 162 (60), 147 (63), 135 (68), 134 (36), 119 (32), 91 (38), 79 (36), 77 (29)	unknown
R5, R8	*C. longa* EOCL	218 (M+, 4), 120 (44), 119 (37), 111 (24), 105 (89), 93 (18), 91 (35), 85 (11), 83 (100), 77 (29), 55 (27)	*α*-turmerone
R5, R8	*C. longa* EOCL	218 (M+, 33), 121 (14), 120 (100), 105 (18), 93 (5), 92 (13), 91 (21), 83 (36), 79 (5), 77 (14), 55 (11)	*β*-turmerone
R6, R9	*C. longa* EOCL	204 (M+, 25), 161 (55), 133 (42), 120 (31), 93 (57), 91 (74), 77 (47), 69 (100)	*β*-sesquiphellandrene

^a^ The same as in Figure 1.

**Table 2:** Antioxidant ability of four species of *Curcuma*, volatile oil of *C. wenyujin* and *C. longa.*

Samples ^a^	Bands ^b^	Contribution to antioxidant capacityof the sample ^c^ (%)	Antioxidant capacity of the sample(correspond to VC, mg/g)
W1	R1	78	12.1
W2	R1	100	7.5
W3	R1	96	8.6
W4	R1	87	15.8
K1	R2	68	6.6
	R3	32	
K2	R2	57	6.5
	R3	36	
K3	R2	47	10.2
	R3	53	
K4	R2	60	8.8
	R3	35	
P1	R4	53	2.1
P2	R4	49	3.5
P3	R4	45	5.2
P4	R4	41	4.2
L1	R5	59	12.4
	R6	28	
L2	R5	61	10.9
	R6	25	
L3	R5	62	13.1
	R6	27	
L4	R5	65	10.6
	R6	29	
EOCW	R7	100	759.3
EOCL	R8	44	373.1
	R9	56	

^a^ The same as in Table 3 except essential oils of *C. wenyujin* (EOCW) and *C. longa* (EOCL); ^b^ The same as in Figure 1; ^c^ The percentage of peak area to total peaks area of investigated sample scanned at 530 nm after DPPH bioautography assay.

**Table 3:** Investigated rhizomes of four species of *Curcuma.*

Species	Code	Location
*C. wenyujin*	W1, W2	Yueqing, Zhejiang Province, China
	W3	Rui’an, Zhejiang Province, China
	W4	Yongjia, Zhejiang Province, China
*C. kwangsiensis*	K1	Qingzhou, Guangxi Province, China
	K2, K3, K4	Wuming, Guangxi Province, China
*C. phaeocaulis*	P1, P2	Chongzhou, Sichuan Province, China
	P3	Zhoudu, Sichuan Province, China
	P4	Wangdan, Sichuan Province, China
*C. longa*	L1, L2, L3	Chongzhou, Sichuan Province, China
	L4	Shuangliu, Sichuan Province, China
